# Microstructure Design for Fast Lifetime Measurements of Magnetic Tunneling Junctions

**DOI:** 10.3390/s19030583

**Published:** 2019-01-30

**Authors:** Andrés Conca Parra, Frederick Casper, Johannes Paul, Ronald Lehndorff, Christian Haupt, Gerhard Jakob, Mathias Kläui, Burkard Hillebrands

**Affiliations:** 1 Fachbereich Physik and Forschungszentrum OPTIMAS, Technische Universität Kaiserslautern, 67663 Kaiserslautern, Germany; hilleb@physik.uni-kl.de; 2 Sensitec GmbH, 55130 Mainz, Germany; Frederick.casper@sensitec.com (F.C.); Johannes.Paul@sensitec.com (J.P.); Ronald.Lehndorff@sensitec.com (R.L.); Christian.Haupt@sensitec.com (C.H.); 3 Institute of Physics, Johannes Gutenberg University, 55122 Mainz, Germany; jakob@uni-mainz.de (G.J.); Klaeui@uni-mainz.de (M.K.)

**Keywords:** Weibull, sensor, reliability, failure, TMR, MTJ, MgO, tunneling barrier, stress

## Abstract

The estimation of the reliability of magnetic field sensors against failure is a critical point concerning their application for industrial purposes. Due to the physical stochastic nature of the failure events, this can only be done by means of a statistical approach which is extremely time consuming and prevents a continuous observation of the production. Here, we present a novel microstructure design for a parallel measurement of the lifetime characteristics of a sensor population. By making use of two alternative designs and the Weibull statistical distribution function, we are able to measure the lifetime characteristics of a CoFeB/MgO/CoFeB tunneling junction population. The main parameters governing the time evolution of the failure rate are estimated and discussed and the suitability of the microstructure for highly reliable sensor application is proven.

## 1. Introduction

Read sensors based on magnetic tunnelling junctions (MTJs) and on the tunneling magnetoresistance (TMR) effect are the dominant technology in hard disc drives largely displacing giant magnetoresistance (GMR) spin valve systems. The use for conventional magnetic field sensors for position, orientation or speed measurement is also fast evolving for industrial applications in the automotive branch, in robotics, current sensing and in machine automation in general. MTJ-based sensors possess two major advantages compared to still widely employed AMR (anisotropic magnetoresistance) and GMR technologies. First, they show a much higher sensitivity due to larger signal amplitude. Technologically, the larger signal amplitude is helpful to eliminate the requirement of a signal preamplifier with the subsequent simplification of the sensing unit design. Secondly, the presence of an insulating layer results in larger resistance values, which make them promising candidates for green energy applications with low-consumption requirements, due to the reduction of the current needed to obtain a voltage signal. Consequently, the main sensor producers are developing new prototypes and products based on MTJs [1,2,3].

Although the clear advantages are pushing for a fast introduction into the market, there is an issue which hinders the implementation of TMR-based devices in many fields, not only in sensorics, but also for other applications as, for instance, memory systems employing spin torque switching [4]. The thin insulating layer responsible for the magnetoresistance effect constitutes also a weak point which is affecting the long term stability against failure of MTJ-based sensor systems. During standard working conditions, the voltage drops over the few nanometer thick tunnelling barrier resulting in a large electrical field (10${}^{7}\;$V/m). This field constitutes a strong stress agent being able, after a certain time, to produce a dielectric breakdown of the barrier and thus a device failure. This process is independent on electrostatic discharge (ESD) events which can also destroy the barrier. While the latter can be prevented with diverting on-chip circuits or by training of the mounting employees, the former cannot be avoided since the stress agent is present any time that the sensor/device is in use. To prove the reliability of sensor use in different fields of application, a fast on-going test method is required.

The lifetime characteristics governing the reliability can only be estimated for a sensor population using a statistical approach. The measurements are performed by setting the sensor elements under stress conditions and waiting for failure events. Since a large number of failure events must be recorded to obtain a reliable statistical population size, the process is extremely time consuming and therefore it is not suitable for on-going production control. Here we present a new microstructure design intended to solve this problem by accelerating the process. The structure design allows for easy upscaling and does not require additional electronics or current controlling diodes.

## 2. Statistics for the Measurement of the Lifetime

The time to breakdown is a statistically distributed parameter and cannot be predicted for a single MTJ since we are here dealing with a stochastic process analogous to metal failure under mechanical stress. The failure rate for a given sensor population and its time evolution characteristics can still be measured and analyzed using statistical methods.

The Weibull statistical distribution is employed for the analysis of the lifetime characteristics of our MTJ populations. This approach can be used in studies of failure events caused by a single stress agent (in our case the electrical field). This approach does not take into account the damages generated by heating or by current-induced electromigration for which other statistical models are more appropriate. For tunneling junctions with low resistance×area (RA) values, the resulting large current density may complicate the measurements.

The Weibull probability density function f(t) describes the fraction of broken elements per time unit: [5,6]

(1) $$f\left(t\right)=\frac{\beta }{\eta }{\left(\frac{t}{\eta }\right)}^{\beta -1}\exp\left(-{\left[\frac{t}{\eta }\right]}^{\beta }\right)$$

Here, η is the characteristic lifetime of the junction population and β is the shape parameter. For measurements, it is more convenient to use the Weibull cumulative function, which describes the total fraction *F* of broken elements after a certain time *t*:

(2) $$F\left(t\right)=1-\exp\left(-{\left[\frac{t}{\eta }\right]}^{\beta }\right)$$

The point t=η, marks the situation at which 63.2% of the elements have already failed. To understand the impact of the shape parameter β in the time evolution of the fraction of failed elements, we distinguish three characteristic cases:

- β<1 Failure rate decreases with time. The population shows a certain degree of infant mortality, i.e., a fraction of the elements breaks shortly after the application of the stress agent. This regime is not desired for production since a large part of the sensor population will fail in an early stage.
- β>1 Failure rate increases with time. The behavior is dominated by *aging*. In addition, an initial time window exists for which the failure rate is negligible. This is the desired situation for applications since it allows the introduction of a guarantee time.
- β=1 Constant failure rate. Transition between the two previous cases. However, it may also be an indication of the existence of an external random agent dominating the behavior when this is always the case (e.g., a defective measurement setup generating peak voltages). The dependence of the lifetime η on the stress agent strength allows to distinguish between both situations.

The suitability of the Weibull distribution for the description of breakdown processes in thin oxide layers [7] and, more concretely, for tunneling devices has been already demonstrated for MTJs with Al${}_{2}$O${}_{3}$ [8,9] and MgO [10,11] barriers.

### Measurement Time

For the measurement of the lifetime η of a MTJ population under a certain level of voltage (stress agent), the single elements must be set continuously under stress for a time span so that enough failure events are recorded, i.e., for a time of the same order of magnitude as the lifetime. This is clearly not feasible for the voltage levels used during standard sensor operation since lifetime values well above 10${}^{6}$ years are common [10]. For this reason, the evolution is accelerated by using large stress levels and measuring the lifetime for different voltage values and using a model for extrapolating to lower voltage values. In the literature several models are chosen, going from an inverse power law model to a more or less complicated exponential law [2,8,9,12]. In our previous work, we have shown that a simple exponential model is suitable for MgO tunneling barriers to explain the dependence on the voltage *V* [10]:

(3) η=Aexp(−BV)

where *A* and *B* are the fit parameters.

To obtain reasonable statistics, a minimum number of elements around 20–30 must be tested per voltage level, given that the voltage is able to generate a failure for most of them. Since, for the required extrapolation, this has to be performed for lifetimes ranging from a few minutes to several hours, typically a total measurement time of around 50–100 h is needed. This prevents an on-going production control or a wide study of parameter influences (such as, for instance, annealing temperature). The time can be shortened by parallel measurement of different junctions if an appropriate needle system is implemented but this has also limitations. One of them is for instance that an ad-hoc system has to be designed and that, due to the space required for the needles, the test junctions will occupy a large area on the production wafer.

## 3. Description of the Microstructure Design

**Figure 1:**
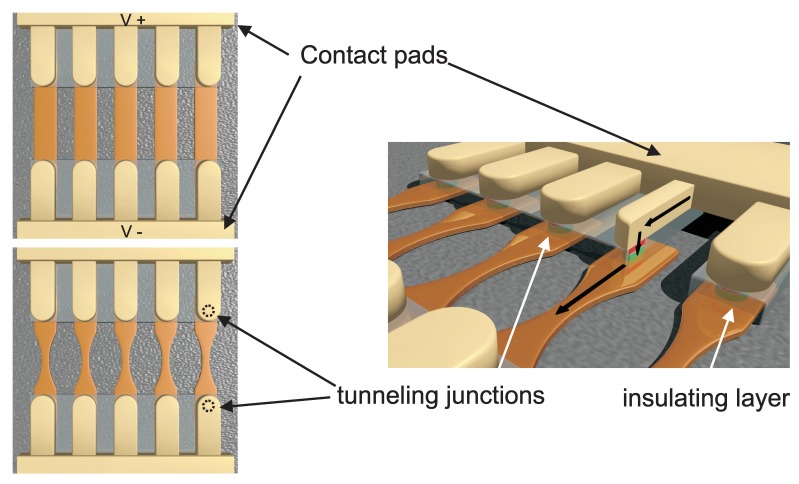
Schematic drawing of the microstructure for fast lifetime measurements. The images on the left show a top view of the two alternative designs with wide (W) and narrow (N) fuse layer. The dashed circles marked the position of the buried junctions. The right image show a side view where a section has been removed to show the junction area. The black arrows indicate the current flow. The drawings are not to scale.

The microstructure design consists of several current lines connected in parallel as shown in Figure 1. The left images show a top view of two alternative designs. On the side view on the right, one of the lines is graphically cut and the current flow is shown as black arrows. The thick current lines (golden colored) are attached to a large contact pad and are deposited on top of an Al${}_{2}$O${}_{3}$ insulating layer with windows that allow for the contact of the top layer of circular shaped MTJs. A much thinner (a few nanometers) metallic layer (orange colored) contacts the bottom layer of the tunneling junctions and it is intended to act as a fuse. In the configuration used for this paper, two tunneling junctions are connected in series in order to increase the resistance of the complete structure before any failure event has occurred.

When the first failure event takes place in one junction of one of the serially connected pairs, the other one is also destroyed immediately because the voltage increases fast by a factor of two. When this happens a short circuit is created between the contact pads (V+ and V−) and a high current flows through this line. The current density in the thin layer is so high that the heat development destroys it fast. The alternative design includes an additional width constriction in order to further increase the current density and accelerate the destruction. After the line is burnt, the voltage applied on the other current lines is recovered and the measurement proceeds. In the following the two alternative designs will be referred as W, for wide fuse line, and N, for narrow line.

The images in Figure 1 are not to scale. The width of the fuse layer is set to 15 μm and it is reduced to 5 μm in the constriction. The length is 35 μm. The MTJ elements are circularly shaped with a diameter of 10 μm. The distance between the current lines is set to 140 μm.

The structure is scalable to a larger number of elements, limited only by the available current source since the total current will increase with the number of lines. The structure is compact and the density of lines can be further increased by reducing the line distance to the limit allowed by the lithographic process. Due to the scalability and compactness, it can be copied on every chip of a sensor wafer allowing also for measurement of lifetime distribution in wafers. A spatial distribution may be present due to the dependence of the lifetime on the barrier thickness [10].

## 4. Results and Discussion

### 4.1. Single Measurements

**Figure 2:**
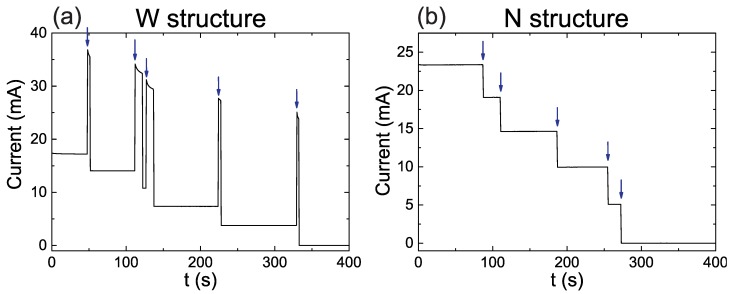
Exemplary single stress measurements for a voltage level of 3.15 V. (**a**,**b**) show the cases for the structure with wide (W) and narrow (N) fuse layers.

Figure 2a,b shows exemplary single stress measurements for a W and a N structure, respectively. Both data sets were collected under voltage levels of 3.15 V, i.e., 1.575 V per tunneling element. The blue arrows mark the position of the failure events, five for each structure. The behavior of both structures differ. In the W structure, each failure event is followed by a large increase of the current. This is due to the fact that the fuse line does not burn immediately and therefore the total resistance of the structure drops. When the layer finally burns, the current decreases to a value lower than the one previous to the failure event, since now the total resistance is increased by the absence of a current line. The time required for the destruction of the fuse layer increases up to 10 s in this example but times as along as 60 s have been also observed. In the N structure, the constriction allows for a much faster destruction of the fuse layer which happens in a time scale below the time resolution of this measurement (0.8 s). With other measurements with better time resolution it was shown that the time required for the destruction of the fuse layer is smaller than 0.2 s.

Further information can be obtained from the size of the current steps in Figure 2b. In ideal conditions, all the steps should have the same height. However, small differences produced during the lithography may introduce minor variations. The size also serves as a control for the reproducibility of the fabrication of the MTJ circular microstructures, the effect of redeposition of metal on the edges during the etching process can easily result in big differences. In the example shown in Figure 2a the current step size is 4.7 mA with a standard deviation of 0.3 mA, proving a large homogeneity in properties.

### 4.2. Weibull Plots and Extrapolation

**Figure 3:**
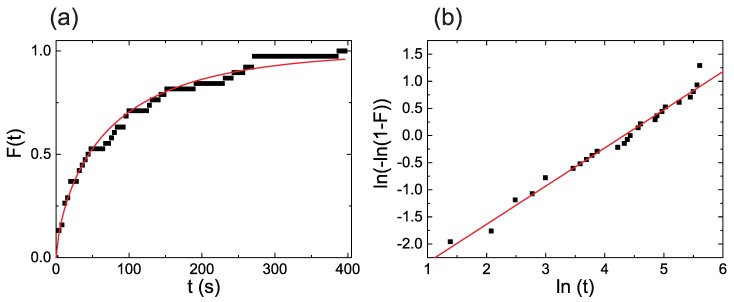
(**a**) Weibull cumulative function measured for a voltage level of 3.2 V with the structure W. The red line is a fit to Equation (2); (**b**) Weibull plot showing the linear behavior expected from Equation (4). The red line is a linear fit.

Figure 3 a shows the measured Weibull cumulative function obtained exemplary for structure W and a voltage level of 3.2 V. The data correspond to a population of 40 elements. The red line is a fit to Equation (2) yielding a lifetime η of 75.5 s. For better comparison between different data sets, the use of Weibull plots is very common and recommended. Applying the logarithmic function to (2) results in:

(4) $$\ln\left[-\ln(1-F(t))\right]=\beta \cdot \ln\left(t\right)-\beta \cdot \ln\left(\eta \right)$$

which is a simple linear dependence for the quantity $\ln\left[-\ln(1-F(t))\right]$ on ln(t). An additional advantage of this expression is that, as shown in Figure 3b, it is very easy to recognize any deviation from the model.

**Figure 4:**
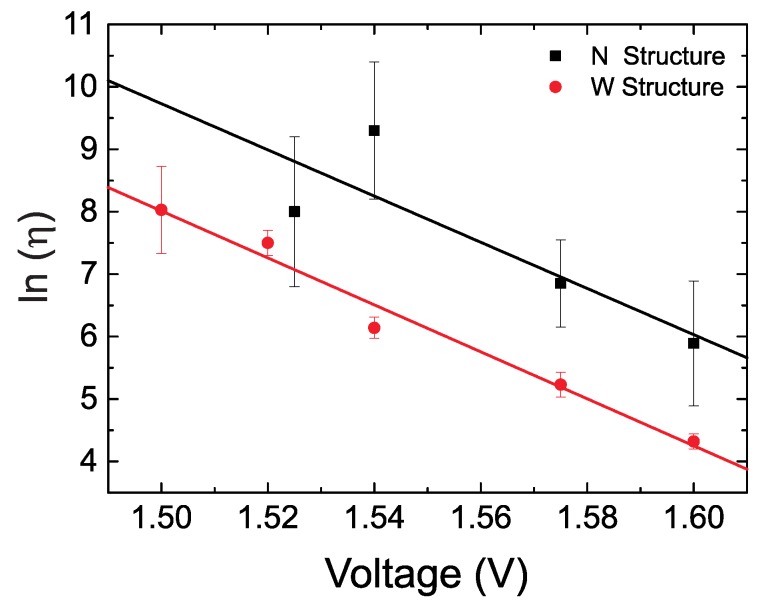
Dependence of the logarithm of the lifetime η on the applied voltage. The data for both alternative designs are shown separately. The lines represent a fit to the exponential law shown in Equation (3).

Figure 4 shows the dependence of the ln(η) on the applied voltage through one junction. The data sets corresponding to measurements with the W and the N structures for the same wafer are shown separately. The lines are a fit to the simple exponential law presented in Equation (3). It can be recognized that both data sets are shifted with respect to each other. This is due to the different resistance value of the fuse layer in structures W and N due to the presence or absence of the constrictions. Since the total voltage is the same, this means that the voltage drop through the MTJ is slightly different. The difference in both fits can be interpreted as a voltage shift of the order of 0.045–0.05 V. The larger error bars obtained for the N structure for the same voltage values is due to the larger lifetime (please note again the logarithmic scale) and the consequently much lower number of recorded failure events in the available number of structures on the wafer.

Despite the shift, the extrapolated lifetimes for V = 0.4 V are 2.2 × 10${}^{12}$ years and 6.2 × 10${}^{12}$ years for the W and N structure, respectively, which are in the same order of magnitude and are comparable to the ones obtained for the same barrier thickness in [10]. The values here are obtained taking into account the error bars due to the linear fit and choosing the worst case scenario. i.e., shortest lifetime. Concerning the β values, we obtain 0.77 ± 0.05 and 0.74 ± 0.09 for the W and N designs, respectively. Both values are the same considering the error bars and point to a small but not vanishing degree of infant mortality in this population.

A comment has to be made concerning the minimal ratio between the resistance of the tunneling junction R${}_{\mathrm{junction}}$ and the resistance of the fuse R${}_{\mathrm{fuse}}$ required to give reliable results. The main requirement is that, once a tunneling junction breaks, the current density increases to a level that assures the burning of the fuse, this is only possible if $R_{\mathrm{junction}}>>R_{\mathrm{fuse}}$. The main mechanism responsible for the destruction of the fuse is thermal due to joule heating caused by the large current density. This will fail at some point if the ratio $R_{\mathrm{junction}}/R_{\mathrm{fuse}}$ is reduced, but the exact value will depend not only of the microstructure itself but also, on the thermal conductivity of the substrate and layers below and above (if present). The thermal conductivity will vary strongly depending if an oxide or silicon substrate is used, and in the last case, it will vary strongly with the doping and thickness. Therefore, the answer to the question needs to be found for each kind of substrate.

With the obtained (η,β) values it is possible to use (2) to extract the expected failure rate for our junctions after a certain elapsed time. The obtained values are below 1 ppm for the measured wafers after 10 years of continuous operation. The very small observed failure rates demonstrate the high reliability of the potential sensors fabricated using these tunneling junctions.

## 5. Conclusions

The data shown here proves that the presented new microstructure designs allow for a fast measurement of the lifetime properties of MTJs employing the Weibull statistical distribution. Parallel application of stress on MTJs enables the study of the failure events and the temporal evolution. The microstructure tested here is limited to five junctions but the design allows for upscaling to an arbitrary number of elements. This can be done without losing the compactness of the design which allows for the placement of the test structure in every sensor chip on a wafer. The parameters governing the time evolution of the sensor reliability against failure, i.e., lifetime η, shape parameter β and the failure rate after a defined elapsed time, can be fast measured with the design, as demonstrated with standard CoFeB/MgO/CoFeB tunneling junctions.

## 6. Materials and Methods

The characterization of the breakdown processes was carried out for circular CoFeB/MgO/CoFeB junctions with a diameter of 10 μm. The barrier is deposited by RF-sputtering and a synthetic antiferromagnet layer structure is used. The thickness of the tunneling barrier is 1.8 nm. SiO${}_{2}$ wafers passivated with an amorphous Al${}_{2}$O${}_{3}$ layer have been used. The deposition and the optical lithography process to create the structures and the copper contact lines used for measuring were carried on in the industrial facilities of Sensitec GmbH [13].

## Abbreviations

The following abbreviations are used in this manuscript:

MTJ	Magnetic tunneling junction
GMR	Giant Magnetoresistance
TMR	Tunneling Magnetoresistance
